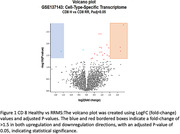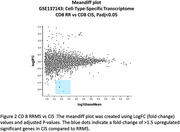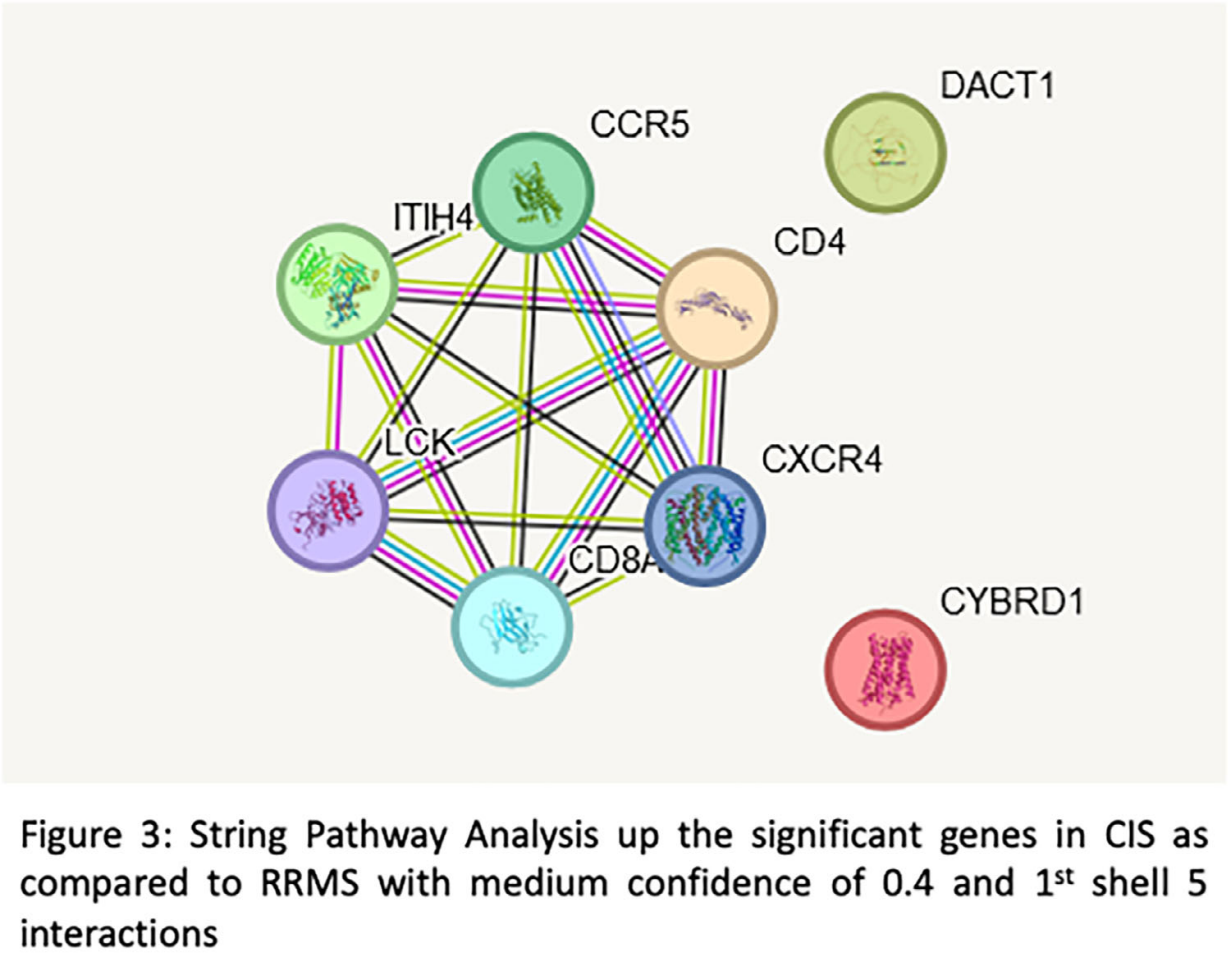# Analysing the Genetic Mosaic of Relapsing Remitting vs Clinically isolated syndrome Multiple Sclerosis: A Bioinformatics Perspective on Subtype‐Specific Variations

**DOI:** 10.1002/alz.093046

**Published:** 2025-01-03

**Authors:** Aman Milind Bhonsale, Ishita Varma, Atharva Subhedar, Shreya Khankar, Snehal Chandak, Mahima Suryawanshi, Siddhali Mast, Tapesh Lalotra, Parth Phadke, Sarthak Pakhmode, Satyendra Tripathi, Vishwajit Deshmukh, Ashlesh Patil

**Affiliations:** ^1^ All India Institute of Medical Sciences, Nagpur, Nagpur, Maharashtra India; ^2^ Research Peer Network ‐ Neurology Study Group, Lucknow, Uttar Pradesh India; ^3^ Bioinformatics Data Analysis Unit (BDAU), AIIMS Nagpur, Nagpur, Maharashtra India

## Abstract

**Background:**

Multiple Sclerosis (MS) is a chronic, etiologically complex disease of the central nervous system (CNS) characterized by inflammation, demyelination, and neuronal damage. MS has seven categories based on disease course. Seventy to eighty percent of individuals with MS initially develop a clinical pattern with periodic relapses and remissions, called relapsing‐remitting MS (RRMS). Clinically isolated syndrome (CIS) refers to a single clinical inflammatory attack on the CNS. Evidence suggests that CD8+ T cells play a substantial role in MS pathophysiology. In this bioinformatic study, we aim to identify specific genes and pathways up‐regulated or down‐regulated in CD8+ T cells of RRMS and CIS subtypes.

**Method:**

The GSE19285 dataset on the Gene Expression Omnibus (GEO) consists of the whole transcriptome of sorted T cells (CD8+) from treatment‐naive MS patients. Age‐matched sampling maintained uniformity. Data analysis used GEO2R with the Benjamini & Hochberg method (False discovery rate, padj < 0.05), and log fold change threshold greater than 1.5 or less than 1.5. We explored genetic profiles in immune cells, comparing RR (n = 17, age 30.76±7.05 years), CIS (n = 17, age 30.76±6.90 years), and healthy controls (n = 17, age 30.71±7.09 years). Gene pathway and protein‐protein interaction analyses of differentially expressed genes used the STRING platform.

**Result:**

The study revealed upregulation of TRBV4‐2 gene in CD8+ cells of RRMS patients compared to healthy controls, while several genes, including AATK, PPP1R14C and TRIM36, were downregulated in RRMS. Two genes, DACT1 and CD4, were identified as upregulated in CD8+ cells, while several genes, including MYOM2, AATK, GPR27, LOC105370088, TRR‐ACG1‐2, KIR2DL3, and SIGLEC10, were downregulated in CIS compared to healthy controls. Additionally, CD4, DACT1, and CYBRD1 genes were up‐regulated in CIS compared to RRMS.

**Conclusion:**

In RRMS, TRIM36’s role in proteostasis and neurodegeneration is highlighted, with protein‐protein interaction analysis showing dysfunctional ubiquitination. Altered antigen processing and presentation in CIS compared to healthy individuals and RRMS suggest differences in pathophysiology in this subtype. Furthermore, recent study has suggested CYBRD1 as a potential MS biomarker.